# The effects of recreational cannabis use on glycemic outcomes and self-management behaviours in people with type 1 and type 2 diabetes: a rapid review

**DOI:** 10.1186/s13643-020-01411-9

**Published:** 2020-08-17

**Authors:** Caroline J. Porr, Patricia Rios, Harpreet S. Bajaj, Aoife M. Egan, Celine Huot, Ryan Batten, Lisa Bishop, Devonne Ryan, Erin Davis, Nazia Darvesh, Arifur Rahman, Shabnam Asghari, Lily Acheampong, Andrea C. Tricco

**Affiliations:** 1 Memorial University of Newfoundland, 300 Prince Philip Drive, St. John’s, NL A1B 3V6 Canada; 2 Li Ka Shing Knowledge Institute, St. Michael’s Hospital, Unity Health Toronto, 209 Victoria Street, East Building, Toronto, ON M5B 1W8 Canada; 3 LMC Diabetes & Endocrinology, 2979 Bovaird Drive E, Brampton, ON L6S 0C6 Canada; 4 Leadership Sinai Centre for Diabetes, Mount Sinai Hospital, 600 University Ave, Toronto, ON M5G 1X5 Canada; 5 Department of Endocrinology, Mayo Clinic Rochester, 200 First Street SW, Rochester, MN 55905 USA; 6 Evidence Synthesis Ireland, National University of Ireland Galway, University Rd, Newcastle, Galway, H91 TK33 Ireland; 7 Department of Pediatrics, Service of Endocrinology, CHU Sainte-Justine, 3175 Côte Sainte-Catherine, Montréal, QC, H3T 1C5 Canada; 8 Epidemiology Division, Dalla Lana School of Public Health, University of Toronto, 155 College St, Toronto, ON M5T 3M7 Canada; 9 Institute of Health, Policy, Management, and Evaluation, University of Toronto, 4th Floor, 155 College St, Toronto, ON M5T 3M6 Canada; 10 Queen’s Collaboration for Health Care Quality, Joanna Briggs Institute Centre of Excellence, Queen’s University, Kingston, ON, Canada

**Keywords:** Cannabis, Diabetes metabolic factors, Diabetes self-management, Type 1 diabetes, Type 2 diabetes, Knowledge synthesis

## Abstract

**Background:**

Recent surveys of Canadian cannabis users reflect increasing consumption rates, some of whom may have diabetes. However, healthcare providers have limited information resources on the effects of recreational cannabis in people with diabetes. This rapid review was commissioned by Diabetes Canada to synthesize available evidence to guide recommendations for care of people 13 years of age and older who live with diabetes.

**Methods:**

PubMed, Embase and PsycINFO databases were searched from January 2008 to January 2019. Study selection, data abstraction and quality appraisal were completed by pairs of reviewers working independently and discrepancies were resolved by a third reviewer with pilot tests completed before each stage to ensure consistency. Data collected from included studies were tabulated and summarized descriptively.

**Results:**

The search resulted in 1848 citations of which 59 publications were selected for screening, resulting in six observational studies (2 full-text articles and 4 conference abstracts) that met the pre-defined criteria for inclusion. Five studies reported higher glycated hemoglobin (HbA1c) in people with type 1 diabetes (T1D) who consumed recreational cannabis. In one study, students aged 17 to 25 years living with T1D self-reported poorer glycemic control and higher HbA1c when smoking cannabis. In one study of adults with T1D, cannabis use within the previous 12 months was associated with almost double the risk of diabetic ketoacidosis compared with no cannabis use (odds ratio [OR] 1.98; confidence interval [CI] [95% CI] 1.01–3.91). Risks for peripheral arterial occlusion and myocardial infarction were found to be higher in people with type 2 diabetes (T2D) who consumed recreational cannabis, and worse renal parameters were also reported in two separate studies of T1D and T2D.

**Conclusions:**

Recreational cannabis use may negatively impact diabetes metabolic factors and self-management behaviours in people with T1D. In people with T2D, recreational cannabis may increase risks for peripheral arterial occlusion, myocardial infarction and renal disease. However, the evidence base of this rapid review was limited to six observational studies of poor to fair methodological quality, and thus, further robust, higher quality research is required to confirm the potential impact of cannabis on diabetes.

**Systematic review registration:**

PROSPERO CRD42019122829

## Background

The *Cannabis Act* came into effect in Canada on October 17, 2018, allowing citizens 19 years of age and older (18 years and older in Alberta and Quebec) to consume cannabis for recreational purposes. Over the past 6 years, consumption of recreational cannabis has increased, overall, with those aged 15 to 24 years consistently showing the highest rates of use according to reports by the National Cannabis Survey [1–4]. Furthermore, even before legislation, 19% of survey respondents indicated that they anticipated using cannabis in 2019, 4% higher than reported consumption rates in 2018, and up to 30% of current cannabis users anticipated increasing their cannabis use in the next year [5].

Recreational cannabis, whether inhaled, brewed or eaten, is typically described by the delta-9-tetrahydrocannabinol (THC) and cannabidiol (CBD) content. THC and CBD are the cannabis cannabinoids known for their pharmacological effects although cannabis contains more than 700 different chemical compounds. Psychotropic effects are attributed to THC while CBD is considered to be more active medicinally, causing little or no feelings of euphoria or impairment [6]. Regardless of whether cannabis is obtained for medicinal or recreational purposes, THC acts by binding to cannabinoid receptors which are distributed in various tissues throughout the body. THC acts as a cannabinoid receptor agonist with no marked preference for either the cannabinoid type 1 receptor (CB1) or cannabinoid type 2 receptor (CB2) [6]. CB1 receptors are localized mostly in the central nervous system, adipose tissue and most endocrine organs, whereas CB2 receptors are mainly present in the peripheral nervous system and immune cells [7]. It should be noted that the concentration of THC in the cannabis products available for recreational use today have been reported to range from 15 to 20% of weight which is significantly higher than in illicit cannabis products consumed 20 years ago [8]. CBD does not have a strong interaction with CB1 and CB2 receptors, but rather has multiple other targets throughout the body such as the transient receptor potential cation channel subfamily V member 1 (TRPV1) nociceptive channels enabling modulation of peripheral hyperalgesia [9].

Given that approximately 2 million Canadians reported having a diagnosis of diabetes in 2017 and that 2 million more new cases may be diagnosed by 2021, adding at least $15 billion in direct healthcare costs, one can expect that a growing number of recreational cannabis users could be people living with diabetes [10, 11]. However, there is limited published literature about the potential link between cannabis use and the incidence of diabetes. Cannabinoids may have the potential to exert physiological effects related to diabetes and glucose control. For example, in human and animal studies, stimulation of CB1 receptors in the insulin-secreting pancreatic beta cells has caused beta cell death [12] and activation of the same receptors in the brain and fat cells has resulted in increased appetite and promotion of adipogenesis and obesity, key risk factors for developing glucose intolerance and type 2 diabetes (T2D) [13]. Consistent with this pharmacological mechanism, researchers found an association between cannabis use and higher caloric intake which may have a negative impact on diabetes metabolic factors and self-management [14].

With both the recreational cannabis use and the prevalence of diabetes expected to rise in the coming years, a thorough review of the currently available evidence on the impacts is warranted. Following the recent enactment of cannabis legislation, Diabetes Canada approached the Strategy for Patient-Oriented Research (SPOR) Evidence Alliance [15] to conduct a review of the scientific literature within a 3-month timeline to examine and appraise existing evidence on the effects of recreational cannabis use in people with diabetes. The results of this timely rapid review were provided to Diabetes Canada and have enabled the publication of a position statement to inform and guide best practices for the care of people with diabetes [16].

## Methods

### Protocol

The protocol and work plan were developed in consultation with Diabetes Canada using the guidance in the Preferred Reporting Items for Systematic Reviews and Meta-Analysis for Protocols (PRISMA-P) [17]. The rapid review protocol was submitted to the PROSPERO database in January 2019 and formally published on March 7, 2019 (CRD42019122829). Although a reporting guideline specific to rapid reviews currently does not exist, we used the Preferred Reporting Items for Systematic Reviews and Meta-Analysis (PRISMA) Statement to guide the reporting of results (Additional file 1: Table S1) [18].

### Eligibility criteria

Eligibility criteria were established in accordance with the Population, Intervention/Exposure, Comparator, Outcome, and Study design (PICOS) framework:

- Population: People aged 13 years or older, with any type of diabetes (type 1, type 2, or gestational) or pre-diabetes
- Intervention/exposure: Consumption of cannabis (all constituents) ingested in any product form (inhaled, brewed, eaten) for recreational purposes
- Comparator: Non-exposure to cannabis
- Outcomes: Metabolic factors related to diabetes (e.g., HbA1c, blood glucose, weight, blood pressure, dyslipidemia), including complications (e.g., [DKA]) or diabetes self-management behaviours (e.g., monitoring blood glucose, taking medication appropriately, exercise, attending medical appointments)
- Study design: Any experimental, quasi-experimental or observational design with the exception of case studies/case series and knowledge syntheses, such as systematic or literature reviews

### Literature search

The search strategy was developed by a librarian (MS) and peer-reviewed by a second librarian (BS) using the PRESS checklist [19]. Database searches were conducted in MEDLINE, Embase and PsycINFO for human studies published in English from January 2008 to January 2019 (see full search strategy in Additional file 2: Table S1). We limited to the past 10 years to provide Diabetes Canada with the most recent literature.

### Study selection, data extraction, quality assessment

The PICOS eligibility criteria were used to develop standardized screening questionnaires prior to level 1 (title/abstract) and level 2 (full-text) screening. Prior to screening at both level 1 and level 2, pilot-tests were carried out with the entire review team (CP, ACT, PR, ND, AE, RB, DR, AR, LA) to refine the screening questions and ensure consistency. At least 70% agreement among reviewers was required to proceed to full screening. Upon successful completion of pilot-testing, pairs of reviewers worked independently to screen titles and abstracts (RB, DR, AR, LA, AE) and full-text articles (RB, DR, AR, LA, AE, PR, ND). Any disagreement between pairs of reviewers was resolved by an independent third reviewer (PR, ND).

A standardized form was developed for data abstraction in consultation with Diabetes Canada that included items related to the study design (e.g., sample size, study setting), participant characteristics (e.g., age, type of diabetes, duration of illness), exposure (e.g., form or frequency of cannabis consumption) and outcomes (e.g., metabolic factors, self-management behaviours). The data abstraction form was pilot-tested by reviewers and once the pilot was successfully completed, pairs of reviewers working independently collecting data from included studies (RB, DR, AR, LA, AE). Discrepancies were resolved consistently by an independent third reviewer (PR).

The quality of included studies was assessed using a modified version of the Newcastle Ottawa Scale (NOS) to evaluate cross-sectional, cohort and case-control studies [20]. The NOS appraises studies based on the methods used to select the study sample, the comparability of groups within a study, and, how exposures and outcomes of interest were assessed. Each item on the NOS is given a letter score with ‘A’ indicating highest quality and descending quality by letters ‘B’, ‘C’, ‘D’ or ‘E’. A pilot-test of the quality appraisal tool was conducted with the review team prior to completing the appraisal and the remaining articles were appraised by pairs of reviewers working independently (RB, DR, AR, LA, AE). Quality appraisal discrepancies were resolved consistently by an independent third reviewer (PR).

### Data synthesis

Data collected from the included studies were synthesized descriptively and presented in summary tables to provide a high-level overview of the available evidence and the quality of the evidence. A meta-analysis was initially considered; however, due to the small number of studies, the high degree of heterogeneity in outcomes and the lack of detailed outcome information available (e.g., effect estimates not provided), quantitative synthesis was not possible.

## Results

### Description of studies

**Fig. 1:**
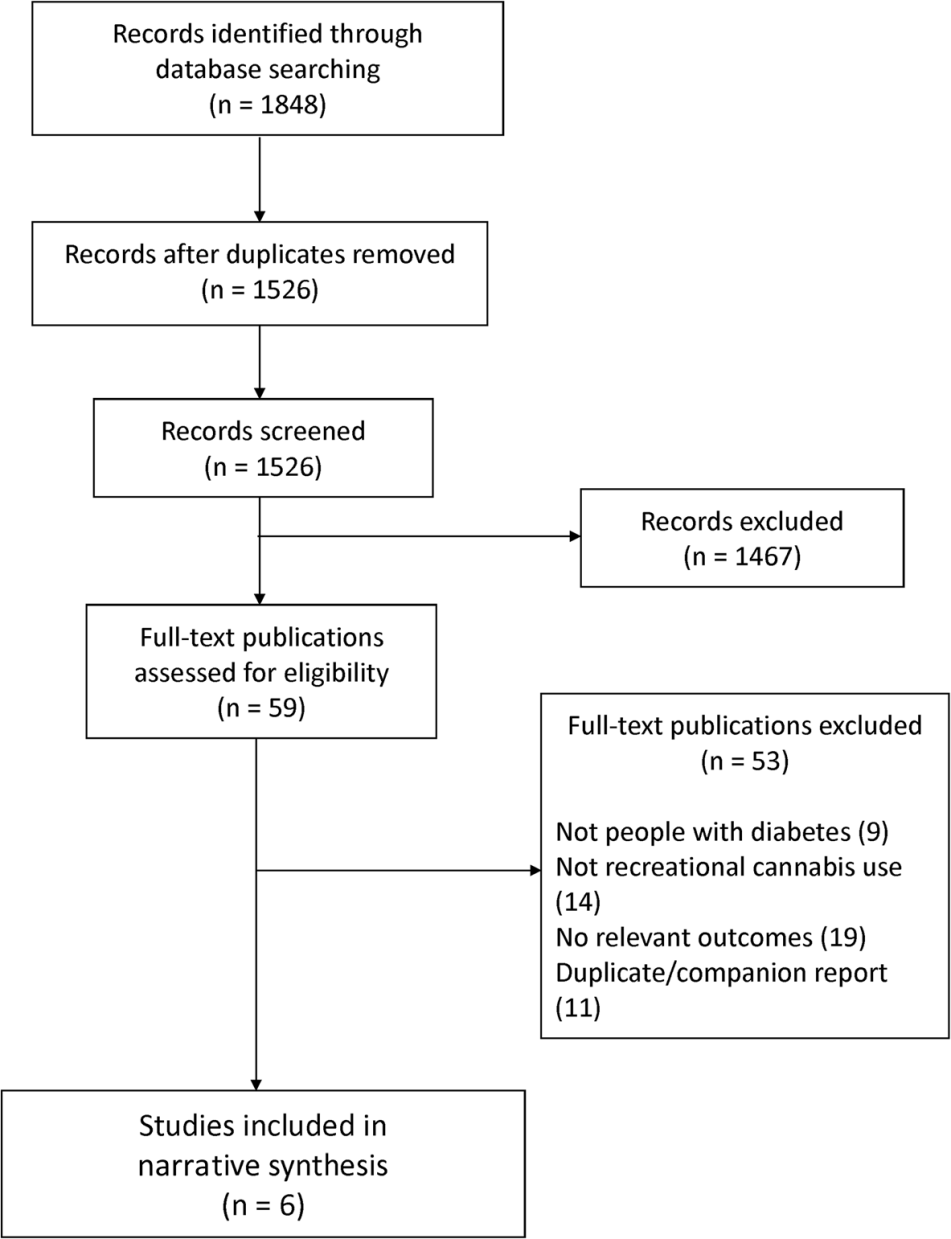
Study flow diagram

The literature search resulted in 1848 articles (Fig. 1). After removing 322 duplicates, 1526 title and abstract citations were included for level 1 screening. Of these, 59 were considered potentially relevant publications for further review. After level 2 screening, two articles and four conference abstracts were included for data synthesis (Fig. 1). The study characteristics of the six included articles are summarized in Table 1. Study designs were cross-sectional (*N *= 4, 67%) [21, 23, 24, 26], cohort (*N* = 1, 17%) [22] and case-control (*N* = 1, 17%) [25]. All studies were published between 2016 and 2018, and the majority were conducted in the USA (*N* = 4, 67%) [21, 22, 24, 25]. The two remaining were from Canada (*Nn* = 1, 17%) [26] and Poland (N*n* = 1, 17%) [23]. Settings included single site (*N* = 2, 33%) [21, 22], multisite (*N* = 1, 17%) [23], city-wide (*N* = 1, 17%) [25], multinational (*n* = 1, 17%) [26] and not reported (*N* = 1, 17%) [24].

**Table 1:** Study characteristics

First author, year	Country	Study design	Setting	Study period	Funding source
Akturk, 2019 [21]	USA	Cross-sectional	Single site	June 2017 and January 2018	Not reported
Helgeson, 2016 [22]	USA	Cohort	Single site	Not reported	National Institutes of Health
Hogendorf, 2016 [23]	Poland	Cross-sectional	Multiple sites	May to June 2013	Medical University of Lodz
Thurheimer-Cacciotti, 2017 [24]	USA	Cross-sectional	Not reported	Not reported	American Diabetes Association
Winhusen, 2018 [25]	USA	Case-control	City-wide	Not reported	Not reported
Wisk, 2018 [26]	USA; Canada	Cross-sectional	Multinational	Not reported	Not reported

Population characteristics are highlighted in Table 2. Sample sizes ranged from 75 to 1184 participants. The average age of participants was 16.5 to 39.1 years; one study by Hogendorf et al. included only teenage participants (15–18 years of age) while two studies included a mix of teenagers and adults [24, 26]; two studies included only adults (> 18 years of age) [21, 22] and one study did not report the age of the sample population [25]. Five of the six studies reported gender distributions ranging between 40.2 and 100% female participants [21–24, 26]. Five of the six studies included investigation of T1D (*n* = 5, 83%) [21–24, 26] while one study examined T2D (*N* = 1, 17%) [25]; no relevant studies that examined populations with gestational diabetes or pre-diabetes were identified. The duration of diabetes ranged from 6.5 to 16.3 years. Only one study indicated complications of diabetes present at baseline: Helgeson et al. reported that 17% of participants had indications of neuropathy from vibratory thresholds greater than age-specific thresholds and 7% had uACR above 30 mg/g. Only three studies cited types of diabetes treatments used by participants: one study reported continuous glucose monitoring and insulin pump use [21]; another reported a combination of insulin injections and insulin pump therapy [22]; and Wisk et al. [26] reported use of insulin pumps, blood sugar testing at least 5 times a day and completing at least 3 HbA1c tests in the past year [26].

**Table 2:** Population characteristics

First author, year	Sample size, mean age (SD), gender/sex distribution^**a**^	Type of diabetes; duration of illness^**b**^ [years, mean (SD)]	Diabetes treatment	Comorbidities/complications	Exclusion criteria
Akturk, 2019 [21]	Group 1: 134, 31.3 (11.1), 55.2% female Group 2: 316, 39.1 (14.2), 40.2% female	Group 1: TID; 16.3 (–) years Group 2: TID; 20.9 (–)	Continuous glucose monitoring: 45.% Group 1, 55.1% Group 2 Insulin pump: 50.7% Group 1, 66.5% Group 2	–	Patients with diabetes other than T1D, pregnancy, and repeat follow-up visits within the study duration
Helgeson, 2016 [22]	132, 23 (–), 56% female	TID; 15.8 (--)	Insulin (units/day): median 50 (IQR 30-65) Insulin pump therapy: 79/183 (43%)	Indications of neuropathy (vibratory thresholds above age-specific norms): 17%; uACR above 30 mg/g: 7%	–
Hogendorf, 2016 [23]	209, 16.5 (1), 48.8% female, 51.2% male	TID; 6.5 (4.4)	–	–	Age 15–18 years
Thurheimer-Cacciotti, 2017 [24]	75, 24.3 (4.1), 100% female	TID–	–	–	–
Winhusen, 2018 [25]	1184 – (–), –	T2D –	–	–	–
Wisk, 2018 [26]	138 20.5 (1.5) 80.40% female	T2D, 10.9 (5.2)^^^	Insulin pump users: 84.1% Testing blood sugar > 5 times/day: 44.2% > 3 HbA1c tests in the past year: 65.9% Average last HbA1c: 7.6	–	–

*SD* standard deviation, *IQR* interquartile range, *T1D* type 1 diabetes, *uACR* urinary albumin/creatinine ratio, *HbA1c* glycated hemoglobin

^a^Values are provided as reported in the studies; calculations for missing values have not been conducted to avoid assumptions regarding the gender distribution of study samples

^b^All studies reported the mean duration of illness except where indicated by ^^^which reported the age at diagnosis [years, mean (SD)]

Cannabis exposure and outcomes varied between studies and are summarized in Table 3. Forms of consumption were explicitly reported in only two studies (*N* = 2, 33%) [21, 22]. One study simply reported that participants used inhaled or smoked cannabis [22] while the other had a more detailed breakdown indicating smoking (*n* = 97, 72.4%), edibles (*n* = 65, 48.5%), vaporization (*n* = 54, 40.3%) and other forms of consumption (*n* = 19, 14.2%) [21]. Only one study reported quantity and frequency of cannabis consumed: Akturk et al. [21] reported cannabis consumption as once a month (*n* = 48, 35.8%), 2–4 times a month (*n* = 14, 10.4%), 2–3 times a week (*n* = 17, 12.7%), and > 4 times a week (*n* = 54, 40.3%). Several outcomes were reported across studies. HbA1c was reported in 83% of the studies (*n* = 5) [21–24, 26]. Other outcomes included DKA (*N* = 1) [21], episodes of severe hypoglycemia (*n* = 1) [21], uACR (*n* = 1) [22], diabetic renal disease (*n* = 1), [25] myocardial infraction (*n* = 1) [25], peripheral arterial occlusion (*n* = 1) [25] and diabetes self-management (*n* = 1) [26].

**Table 3:** Exposure characteristics and outcomes

First author, year	Consumption method; description	Quantity and frequency consumed	Outcome name	Results
Akturk, 2019 [21]	Group 1: multiple Smoking: 97 (72.4%) Edible: 65 (48.5%) Vaporization: 54 (40.3%) Other: 19 (14.2%) Group 2: not applicable (non-users)	Group 1: < 1 time/month: 48 (35.8%) 2–4 times/month: 14 (10.4%) 2–3 times/week: 17 (12.7%) > 4 times/week: 54 (40.3%) Group 2: not applicable (non-users)	Outcome 1: risk of DKA	Cannabis use within the previous 12 months was associated with an increased risk of DKA compared with no cannabis use (entire cohort OR 1.98, 95% CI 1.01–3.91)
			Outcome 2: HbA1c mean level	Group 1: HbA1c: 8.4% (SD 2.0) [*p* < .01] Cannabis users had a mean 0.41% higher HbA1c level than nonusers when adjusted for insulin delivery method, income and age (95% CI, 0.38-0.43) Group 2: HbA1c: 7.6% (SD 1.6) [*p* < .01]
			Outcome 3: episodes of severe hypoglycemia	Group 1: 15.6% (21 of 134) [*p* = .17] Group 2: 20.3% (64 of 316) [*p* = .17]
Helgeson, 2016 [22]	Inhaled; smoked cannabis	–	Outcome 1: HbA1c level	Average HbA1c was 8.8% (12% with HbA1c > 11%) [at baseline] Smoking cannabis was related to higher HbA1c (*r* = .30, *p* < 0.01)
			Outcome 2: albumin-to-creatinine ratio	Smoking cannabis was related to higher uACR (*r* = .22, *p* < 0.05)
Hogendorf, 2016 [23]	–	–	Outcome 1: glycemic control	“Half of the [T1D] patients (53%) had HbA1c levels above 8% [at baseline]; lifetime and last 12-month use of cannabis were associated with poorer glycemic control (HbA1c ≥ 8%), *p* < 0.01 and *p* < 0.02, respectively.”
			Outcome 2: glycemic control	HbA1c of 6-8%: 14/89 tried cannabis HbA1c of 8-10% : 11/62 HbA1c of 10-12%: 9/30 HbA1c >12%: 4/8 tried cannabis (*p* = 0.03)
Thurheimer-Cacciotti, 2017 [24]	–	–	Outcome 1: glycemic control	“Women who reported multiple (> 1) risk-taking behaviours were more likely to have a higher HbA1c (> 8%) compared to women who reported 0–1 risky behaviour (RR = 1.29, 95% CI 0.605–2.742).”
Winhusen, 2018 [25]	–	–	Outcome 1: diabetic renal disease	Cannabis use was associated with a statistically significant increased risk of diabetic renal disease
			Outcome 2: myocardial infarction	Cannabis use was associated with a statistically significant increased risk of myocardial infarction
			Outcome 3: peripheral arterial occlusion	Cannabis use was associated with a statistically significant increased risk of peripheral arterial occlusion
			Outcome 4: neuropathy	Cannabis use was not associated with a statistically significant increased risk of neuropathy
			Outcome 5: cerebrovascular accident	Cannabis use was not associated with a statistically significant increased risk of cerebrovascular accident
Wisk, 2018 [26]	–	–	Outcome 1: diabetes self-management	“Much like their peers, college students with T1D frequently consume alcohol and cannabis; those with T1D who use more frequently experience higher HbA1c and are less likely to achieve glycemic targets, independent of blood glucose testing and diabetes burden.”
			Outcome 2: most recent HbA1c level	“Multivariable analyses revealed that those who drank 3+ days in the past month (50.7% of sample) had significantly higher HbA1c (by 0.63%, *p* < 0.01) and were significantly more likely to be above recommended glycemic targets (OR 3.08, 95% CI 1.59–5.98). Similar results were observed for cannabis and cigarettes.”

*OR* odds ratio, *RR* relative risk, *p p* value, *SD* standard deviation, *95% CI* 95% confidence interval, *DKA* diabetic ketoacidosis, *HbA1c* glycated hemoglobin, *uACR* urinary albumin/creatinine ratio, *T1D* type 1 diabetes

### Quality appraisal results

NOS quality appraisal results are summarized in Table 4. Overall, the quality of the included studies ranged from poor to moderate due to a number of methodological limitations. The quality of the four cross-sectional studies [21, 23, 24, 26], in particular, was limited by a reliance on self-reported data, selective or non-representatively sampled cohorts, and response rates below 75%. The cohort [22] and case-control [25] studies were of slightly better quality, as they used appropriately representative samples, relied on independently assessed outcome data and had adequate follow-up rates or were able to account for missing data.

**Table 4:** Results of quality appraisal with the Newcastle Ottawa Scale

**Cross-sectional studies (*n*** **= 4)**								
**First author, year**	**Is the sample definition adequate?**	**Representativeness of the exposed cohort**			**Ascertainment of exposure**	**Assessment of outcome**	**Adequacy of response rate**	
Akturk, 2019 [21]	B–records/self-report	B–somewhat representative			C–written self-report	A–independent or blind	C–inadequate	
Hogendorf, 2016 [23] Thurheimer-Cacciotti, 2017 [24]	B–records/self-report	B–somewhat representative			C–written self-report	C–self-report	B–small number lost	
	C–no description	C–selected group			D–no description	C–self-report	Unclear	
Wisk, 2018 [26]	B–records/self-report	B–somewhat representative			C–written self-report	C–self-report	D–no description	
**Case-control studies (*n*** **= 1)**								
**First author, year**	**Is the case definition adequate?**	**Representativeness of the cases**	**Selection of controls**	**Definition of controls**	**Comparability of cases and controls**	**Ascertainment of exposure**	**Same method for cases and controls**	**Non-response rate**
Winhusen, 2018 [25]	B–records/self-report	A–consecutive or representative	B–hospital	A–no history	A–age and other factor	A–secure record	A–yes	Unclear
**Cohort studies (*n*** **= 1)**								
**First author, year**	**Representativeness of the exposed cohort**	**Selection of the non-exposed cohort**	**Ascertainment of exposure**	**Demonstration outcome was not present at thestart**	**Comparability of cohorts**	**Assessment of outcome**	**Was follow-up long enough for outcomes to occur?**	**Adequacy of follow-up of cohorts**
Helgeson, 2016 [22]	B–somewhat representative	A–same community	D–no description	A–yes	D–no description	A–independent or blind	A–yes	B–small number lost

### Main findings

### Diabetes-related metabolic factors (including complications)

All of the six studies [21–26] reported on diabetes metabolic factors among cannabis users, with only one study focusing on diabetes self-management behaviours. Five studies [21–24, 26] reported a statistically significant increase in HbA1c among a total of 1004 participants with T1D who consumed cannabis. Akturk et al. (2019) discovered that when adjusting for insulin delivery method, income and age, cannabis users with T1D had a 0.41% higher mean HbA1C level than non-users (8.4% [SD 2.0%] vs 7.6% [SD 1.6%], *p*< .01) but there was no difference in severe hypoglycemia requiring hospitalization (21 of 134 [15.6%] vs 64 of 316 [20.3%], p= .17) among cannabis users with T1D. Akturk et al. also reported a statistically significant increased risk of DKA associated with cannabis use (OR 1.98; 95% CI 1.01–3.91). Hogendorf et al. reported poorer glycemic control (HbA1c ≥ 8%) with lifetime and last 12-month use of cannabis (*p* < 0.01 and 0.02, respectively). Specifically, among cannabis users, 14/89 of all participants had HbA1c levels of 6–8%; 11/62 had HbA1c levels of 8–10%; 9/30 had HbA1c levels of 10–12%; and 4/8 had HbA1c levels > 12% (*p* = 0.03). Helgeson et al. (2016) measured HbA1c in 132 participants with T1D. At baseline, the mean HbA1c level was 8.8% (12% of the sample had an HbA1c > 11%). They concluded that smoking either cigarettes or cannabis was related to higher HbA1c (*r* = .54, *p* < 0.001 and *r* = .30, *p* < 0.01) and higher uACR (*r* = .25, *p* < 0.05 and *r* = .22, *p* < 0.05). Although descriptions of the direct cannabis effects were unclear, Wisk et al. reported that participants who consumed alcohol 3 or more days in the past month, similar to those with cannabis use, had significantly higher HbA1c levels (by 0.63%, *p* < 0.01) and more likely not to achieve their recommended glycemic targets (OR 3.08; 95% CI 1.59–5.98). Similarly, direct cannabis effects were vague in the cross-sectional study by Thurheimer-Cacciotti et al., but among 75 participants with T1D, differences in HbA1c (> 8%) were reported between those who chose risk-taking behaviours (e.g., consuming alcohol or illicit drugs) when compared to participants who reported less risky behaviours (RR 1.29; 95% CI 0.605–2.742).

Complications of diabetes were found among participants with T2D in a case control study involving 1184 participants [25]. Specifically, cannabis users were at a statistically significant risk for peripheral arterial occlusion, myocardial infarction and renal disease. Signs of renal disease (increased uACR) were also present in cannabis users with T1D in a cohort study involving 132 participants [22]. However, cannabis use resulted in no increased risk for neuropathy or cerebrovascular accident (e.g., stroke) in the case-control study of 1184 participants with T2D [25].

### Diabetes self-management behaviours

Wisk, et al. (2018) conducted a cross-sectional study to estimate the impact of substance use (including cannabis) and self-management behaviours on glycemic control in 138 college students with T1D. Students aged 17 to 25 years from universities and colleges across 30 states, Washington D.C., and Canada self-reported their substance use behaviours, diabetes self-management and most recent HbA1c level. The researchers discovered after multivariable analyses that students who smoked cannabis failed to maintain glycemic control and reported higher HbA1c values.

## Discussion

Findings from this rapid review of six observational studies suggest an association between recreational cannabis use and both acute adverse effects (a statistically significant increase in HbA1c, together with an elevated risk for DKA in people with T1D) and chronic complications (elevated uACR in people with T1D and T2D, and, higher rates of peripheral artery occlusion, myocardial infarction and renal disease in people with T2D). In terms of cannabis effects on HbA1c, based on the review, the HbA1c differences between users and non-users with T1D were modest when adjusting for other confounding factors (0.41% in one cross-sectional study). While it is possible that these changes may also occur in patients with T2D, suggesting that chronically elevated glucose values could lead to the observed increased rates of macrovascular complications is uncertain. Indeed, smoking cannabis has been associated with increases in blood pressure, decreases in high-density lipoprotein cholesterol and a lower threshold for developing chest pain among users with angina [27–30]. This suggests an effect on cardiovascular outcomes independent of glycemic parameters. Furthermore, use of cannabis is associated with unhealthy behaviours, including illicit drug use, tobacco smoking and a high caloric diet [31]. It is plausible that these other behaviours could be driving poor metabolic health rather than the direct effects of cannabis. Additionally, it is plausible that recreational cannabis use leads to poorer self-care behaviours (e.g., adequate glucose monitoring, adherence to medications, dietary and physical activity recommendations) which in turn could lead to worse control of cardiovascular risk factors.

Pharmacologically, endogenous cannabinoids are known to regulate energy balance and food intake, and have effects on the limbic system, hypothalamus, adipose tissue and gastrointestinal tract. Cannabinoids are known to cause altered gut motility and can lead to vomiting syndrome in some users [32, 33]. These effects, along with alterations in patient self-management behaviours, may explain the increased risk of DKA noted in the review findings.

In light of the rising prevalence of diabetes and continuing rise in recreational cannabis use, these noteworthy findings have been reported to Diabetes Canada (the commissioning body) but should also be disseminated to other knowledge users in Canada and beyond to facilitate informed practice and decision-making. The *Diabetes Canada Position Statement on Recreational Cannabis Use in Adults and Adolescents with Type 1 and Type 2 Diabetes* was generated based on this review [16]. Diabetes Canada recommends that healthcare providers ask patients about recreational cannabis use without judgment. Cannabis should be considered an important item in the patient’s medical history and a key health information topic for people living with diabetes [16].

Critical, however, to informed decision-making among frontline healthcare providers and people living with diabetes, is an understanding of the limitations of the review findings. Studies available were of poor quality, overall. Four of the six studies were conference abstracts with limited detail, and none of the studies used independent or blind assessments to ascertain exposure and to determine outcomes; additionally, these abstracts likely did not undergo any form of peer review. Also, a recent update of the literature search (January 2020) found new publications on the topic; however, none of the studies were of high quality and did not provide any new evidence that would alter the conclusions of the current review [34–41]. The form of cannabis exposure was reported in only two studies and the frequency was recorded in just one study, based on patient recall. Moreover, without robust randomized controlled intervention studies one cannot discern precisely how quitting recreational cannabis use may improve diabetes metabolic factors, complications and self-management. Additionally, we do not have a timeline for when complications developed relative to cannabis use or if all forms of cannabis exposure (such as inhaled, brewed or eaten) are associated with an increased rate of complications, further precluding assumptions about causality. This review also highlights some important gaps in the literature as no relevant evidence examining populations with gestational or pre-diabetes were identified and only one of the included studies focused on adolescents. Finally, this rapid review was limited in scope, as commissioned by Diabetes Canada, the focus was exclusively on the PICOS framework criteria outlined in the ‘Methods’ section and did not consider broader physical or psychological impacts of cannabis including potential medicinal benefits or general health risks.

Nonetheless, the information from this rapid review may assist healthcare providers to dispense guidance to their patients living with diabetes who use cannabis. Patients should be reminded that the safety of recreational cannabis has not been demonstrated and that there is some evidence to suggest that regular cannabis consumption is associated with (a) worsening of glycemic control, (b) more diabetes-related complications, (c) poorer self-management behaviours (e.g., adequate glucose monitoring, adherence to medications, dietary and physical activity recommendations), and (d) increased risk for DKA among those with T1D. When considering the potential risks, the safest approach is to recommend against the use of recreational cannabis for people with diabetes. For people aged 13 years and older with T1D and T2D who intend to use cannabis recreationally, individualized assessment and counseling should be offered with emphasis on risk reduction. More research is needed to determine the unequivocal effects of recreational cannabis use on diabetes and general health and well-being.

## Conclusions

This rapid review raises important concerns regarding the potential for recreational cannabis use to negatively impact the metabolic health of people with T1D and T2D. While it would be difficult to conduct a randomized controlled trial that compares a cannabis cessation intervention to continued cannabis use, there is clearly a need for more robust studies to further elucidate this association. Moreover, mechanistic studies (including dose-response studies) would enhance understanding of the biological effects of cannabis use in people with and without diabetes. However, if relying on observational students, researchers should more closely assess for confounding variables by referring to guidance documents for methodological recommendations how to mitigate confounders and other potential threats to validity. In the interim, people with diabetes should carefully consider the use of recreational cannabis, particularly those with cardiovascular comorbidities or poorly controlled diabetes who are already at high risk for complications.

## Supplementary information

**Additional file 1:** Table S1 PRISMA checklist of reporting items for a systematic review.

**Additional file 2:** Table S1 Literature search strategy.

## Data Availability

All data generated or analysed during this study are included in this published article.

## Abbreviations

95% CI: 95% confidence interval
CBD: Cannabidiol
CB1: Cannabinoid receptor 1
CB2: Cannabinoid receptor 2
DKA: Diabetic ketoacidosis
HbA1c: Glycated hemoglobin
*N*: Sample size
NOS: Newcastle Ottawa Scale
OR: Odds ratio
*p*: *p* value
PICOS: Population, Intervention, Comparator, Outcome, Study design
PRESS: Peer Review of Electronic Search Strategies
*r*: *r* value: Pearson correlation coefficient
RR: Relative ratio
SD: Standard deviation
SPOR: Strategy for Patient-Oriented Research
T1D: Type 1 diabetes
T2D: Type 2 diabetes
THC: Delta-9-tetrahydrocannabinol
TRPV1: Transient receptor potential vanilloid 1
uACR: Urinary albumin-to-creatinine ratio
