# Supplementary material for: The effects of recreational cannabis use on glycemic outcomes and self-management behaviours in people with type 1 and type 2 diabetes: a rapid review
Source: Syst Rev. 2020 Aug 17;9:187. doi: 10.1186/s13643-020-01411-9 (PMC7433109; doi:10.1186/s13643-020-01411-9)
Supplement: Supplementary file 2 — Additional file 2: Table S1 Literature search strategy. [file 13643_2020_1411_MOESM2_ESM.docx]

PubMed (includes MEDLINE)

Search conducted 09 January 2019

|  | **Query** | **Results** |
| --- | --- | --- |
| #1 | "Diabetes Mellitus"[Mesh] OR diabet*[tiab] OR prediabet*[tiab] OR "type 1 DM"[tiab] OR "type 2 DM"[tiab] OR T1DM[tiab] OR T2DM[tiab] OR IDDM[tiab] OR MODY[tiab] OR NIDDM[tiab] OR "gestational DM"[tiab] | 630,566 |
| #2 | "Cannabis"[Mesh] OR "Cannabinoids"[Mesh] OR "Marijuana Smoking"[Mesh] OR "Marijuana Use"[Mesh] OR "Marijuana Abuse"[Mesh] OR "Medical Marijuana"[Mesh] OR marijuana[tiab] OR marihuana[tiab] OR cannabis[tiab] OR cannabinoid*[tiab] OR tetrahydrocannabinol*[tiab] OR dronabinol*[tiab] OR THC[tiab] OR cannabidiol*[tiab] OR CBD[tiab] OR cannabinol*[tiab] OR "c.indica"[tiab] OR "c.sativa"[tiab] OR bhang[tiab] OR cannador[tiab] OR charas[tiab] OR eucannabinolide*[tiab] OR ganja[tiab] OR hash[tiab] OR hashish[tiab] OR hemp[tiab] OR epidiolex[tiab] OR marinol[tiab] OR "qcd 84924"[tiab] OR syndros[tiab] OR deltanyne[tiab] OR "abbott 40566"[tiab] OR namisol[tiab] OR dronabinolum[tiab] | 52,197 |
| #3 | #1 AND #2 | 599 |
| #4 | "Animals"[Mesh] NOT "Humans"[Mesh] | 4,533,644 |
| #5 | #3 NOT #4 | 468 |
| #6 | #3 NOT #4 (Filter: 2008-present) | 352 |

EMBASE (via Embase.com)

Search conducted 09 January 2019

|  | **Query** | **Results** |
| --- | --- | --- |
| #1 | 'diabetes mellitus'/exp OR diabet*:ab,ti OR prediabet*:ab,ti OR 'type 1 DM':ab,ti OR 'type 2 DM':ab,ti OR T1DM:ab,ti OR T2DM:ab,ti OR IDDM:ab,ti OR MODY:ab,ti OR NIDDM:ab,ti OR 'gestational DM':ab,ti OR 'pregnancy induced DM':ab,ti | 1,042,067 |
| #2 | 'Cannabis (genus)'/exp OR 'cannabinoid'/exp OR 'cannabis use'/exp OR 'cannabis addiction'/de OR 'cannabis-induced psychosis'/de OR marijuana:ab,ti OR marihuana:ab,ti OR cannabis:ab,ti OR cannabinoid*:ab,ti OR tetrahydrocannabinol*:ab,ti OR dronabinol*:ab,ti OR THC:ab,ti OR cannabidiol*:ab,ti OR CBD:ab,ti OR cannabinol*:ab,ti OR 'c.indica':ab,ti OR 'c.sativa':ab,ti OR bhang:ab,ti OR cannador:ab,ti OR charas:ab,ti OR eucannabinolide*:ab,ti OR ganja:ab,ti OR ganjah:ab,ti OR hash:ab,ti OR hashish:ab,ti OR hemp:ab,ti OR epidiolex:ab,ti OR 'gwp 42003p':ab,ti OR gwp42003p:ab,ti OR nabidiolex:ab,ti OR 'ea 1477':ab,ti OR ea1477:ab,ti OR marinol:ab,ti OR 'qcd 84924':ab,ti OR qcd84924 OR syndros:ab,ti OR tetranabinex:ab,ti OR deltanyne:ab,ti OR 'abbott 40566' OR abbott40566:ab,ti OR namisol:ab,ti OR dronabinolum:ab,ti OR 'CCRIS 4726':ab,ti OR CCRIS4726:ab,ti | 88,739 |
| #3 | #1 AND #2 | 2,172 |
| #4 | ('animal'/exp OR 'nonhuman'/de) NOT 'human'/de | 6,727,478 |
| #5 | #3 NOT #4 | 1,829 |
| #6 | #3 NOT #4 (Filter: 2008-present) | 1,438 |

PsycINFO (via EBSCOhost)

Search conducted 09 January 2019

|  | **Query** | **Results** |
| --- | --- | --- |
| #1 | DE "Diabetes" OR DE "Diabetes Mellitus" OR DE "Gestational Diabetes" OR DE "Type 2 Diabetes" OR TI diabet* OR AB diabet* OR TI prediabet* OR AB prediabet* OR TI "type 1 DM" OR AB "type 1 DM" OR TI "type 2 DM" OR AB "type 2 DM" OR TI T1DM OR AB T1DM OR TI T2DM OR AB T2DM OR TI IDDM OR AB IDDM OR TI MODY OR AB MODY OR TI NIDDM OR AB NIDDM OR TI "gestational DM" OR AB "gestational DM" OR TI "pregnancy induced DM" OR AB "pregnancy induced DM" | 29,291 |
| #2 | DE "Cannabis" OR DE "Hashish" OR DE "Marijuana" OR DE "Cannabinoids" OR DE "Tetrahydrocannabinol" OR DE Marijuana Usage OR TI marijuana OR AB marijuana OR TI marihuana OR AB marihuana OR TI cannabis OR AB cannabis OR TI cannabinoid* OR AB cannabinoid* OR TI tetrahydrocannabinol OR AB tetrahydrocannabinol OR TI dronabinol OR AB dronabinol OR TI THC OR AB THC OR TI cannabidiol OR AB cannabidiol OR TI CBD OR AB CBD OR TI cannabinol OR AB cannabinol OR TI "c.indica" OR AB "c.indica" OR TI "c.sativa" OR AB "c.sativa" OR TI bhang OR AB bhang OR TI cannador OR AB cannador OR TI charas OR AB charas OR TI eucannabinolide* OR AB eucannabinolide* OR TI ganja OR AB ganja OR TI ganjah OR AB ganjah OR TI hash OR AB hash OR TI hashish OR AB hashish OR TI hemp OR AB hemp OR TI epidiolex OR AB epidiolex OR TI "gwp 42003p" OR AB "gwp 42003p" OR TI gwp42003p OR AB gwp42003p OR TI nabidiolex OR AB nabidiolex OR TI "ea 1477" OR AB "ea 1477" OR TI ea1477 OR AB ea1477 OR TI marinol OR AB marinol OR TI "qcd 84924" OR AB "qcd 84924" OR TI qcd84924 OR AB qcd84924 OR TI syndros OR AB syndros OR TI tetranabinex OR AB tetranabinex OR TI deltanyne OR AB deltanyne OR TI "abbott 40566" OR AB "abbott 40566" OR TI abbott40566 OR AB abbott40566 OR TI namisol OR AB namisol OR TI dronabinolum OR AB dronabinolum OR TI "CCRIS 4726" OR AB "CCRIS 4726" OR TI CCRIS4726 OR AB CCRIS4726 | 23,834 |
| #3 | S1 AND S2 | 71 |
| #4 | S1 AND S2 (Filter: 2008-present) | 58 |
